# High incidence of hepatocellular carcinoma and cirrhotic complications in patients with psychiatric illness: a territory-wide cohort study

**DOI:** 10.1186/s12876-020-01277-0

**Published:** 2020-04-29

**Authors:** Terry Cheuk-Fung Yip, Grace Lai-Hung Wong, Yee-Kit Tse, Becky Wing-Yan Yuen, Hester Wing-Sum Luk, Marco Ho-Bun Lam, Michael Kin-Kong Li, Ching Kong Loo, Owen Tak-Yin Tsang, Steven Woon-Choy Tsang, Henry Lik-Yuen Chan, Yun-Kwok Wing, Vincent Wai-Sun Wong

**Affiliations:** 1 Department of Medicine and Therapeutics, Prince of Wales Hospital, Hong Kong, China; 2 State Key Laboratory of Digestive Disease, The Chinese University of Hong Kong, Hong Kong, China; 3 Department of Psychiatry, Shatin Hospital, The Chinese University of Hong Kong, Hong Kong, China; 4 Department of Medicine and Geriatrics, Tuen Mun Hospital, Hong Kong, China; 5 Department of Medicine and Geriatrics, Kwong Wah Hospital, Hong Kong, China; 6 Department of Medicine and Geriatrics, Princess Margaret Hospital, Hong Kong, China; 7 Department of Medicine, Tseung Kwan O Hospital, Hong Kong, China

**Keywords:** Liver fibrosis, Liver neoplasms, Liver failure, Mortality, Mental disorders

## Abstract

**Background:**

Because of high-risk behaviours, sedentary lifestyle and side effects of medications, psychiatric patients are at risk of viral hepatitis, alcohol-related liver disease and non-alcoholic fatty liver disease. We aimed to study the incidence of hepatocellular carcinoma (HCC) and cirrhotic complications in psychiatric patients.

**Methods:**

We identified consecutive adult patients in all public hospitals and clinics in Hong Kong with psychiatric diagnoses between year 2003 and 2007 using the Clinical Data Analysis and Reporting System, which represents in-patient and out-patient data of approximately 80% of the 7.4-million local population. The patients were followed for liver-related events (HCC and cirrhotic complications) and deaths until December 2017. Age- and sex-standardized incidence ratio (SIR) of HCC in psychiatric patients to the general population was estimated by Poisson model.

**Results:**

We included 105,763 psychiatric patients without prior liver-related events in the final analysis. During a median (interquartile range) follow-up of 12.4 (11.0–13.7) years, 1461 (1.4%) patients developed liver-related events; 472 (0.4%) patients developed HCC. Compared with the general population, psychiatric patients had increased incidence of HCC (SIR 1.42, 95% confidence interval [CI] 1.28–1.57, *P* < 0.001). The SIR was highest in patients with drug-induced (SIR 3.18, 95% CI 2.41–4.11, *P* < 0.001) and alcohol-induced mental disorders (SIR 2.98, 95% CI 2.30–3.81, *P* < 0.001), but was also increased in patients with psychotic disorders (SIR 1.39, 95% CI 1.16–1.65, *P* < 0.001) and mood disorders (SIR 1.16, 95% CI 1.00–1.34, *P* = 0.047). Liver disease was the fifth most common cause of death in this population, accounting for 595 of 10,614 (5.6%) deaths. Importantly, 569 (38.9%) patients were not known to have liver diseases at the time of liver-related events. The median age at HCC diagnosis (61 [range 26–83] years) was older and the median overall survival (8.0 [95% CI 5.0–10.9] months) after HCC diagnosis was shorter in this cohort of psychiatric patients than other reports from Hong Kong.

**Conclusions:**

HCC, cirrhotic complications, and liver-related deaths are common in psychiatric patients, but liver diseases are often undiagnosed. More efforts are needed to identify liver diseases in the psychiatric population so that treatments and screening for HCC and varices can be provided to patients in need.

## Background

Chronic liver diseases and the resultant portal hypertension and hepatocellular carcinoma (HCC) are among the leading causes of death worldwide [1]. Psychiatric illness is also highly prevalent and accounts for 13% of disability-adjusted life-years globally [2]. Patients with psychiatric illness are at risk of different liver diseases. Alcohol and substance abuse predisposes patients to alcohol-related liver disease and hepatitis C virus infection [3]. Excessive alcohol consumption also aggravates existing liver diseases such as chronic viral hepatitis.

Moreover, non-alcoholic fatty liver disease (NAFLD) affects a quarter of the global adult population and has become one of the leading causes of cirrhosis and HCC in the Western world [4–6]. Patients with psychiatric illness often have obesity and metabolic syndrome [7]. A number of potential mechanisms have been proposed, including activation of the hypothalamus-pituitary-adrenal axis through acute stress response, abnormalities in the autonomic nervous system, systemic inflammation and oxidative stress, and changes in appetite-controlling hormones. Patients with psychiatric illness tend to lead a more sedentary lifestyle. Second generation antipsychotics, antidepressants and mood stabilizers may be obesogenic and/or hepatotoxic [8]. Some genes (e.g. CLOCK genetic variants) affect both metabolism and mental illness [9, 10]. Besides, diabetes, cardiovascular disorders and sleep and/or circadian disruptions are common in psychiatric illness.

Accordingly, a study by the United States Veterans Integrated Services Network showed that 22.4% of patients with schizophrenia had one or more liver diseases [11]. NAFLD patients with depression also have more severe liver histology [12]. However, liver biopsy is performed in a highly selected population, which cannot represent the average patients with psychiatric illness. The magnitude of liver-related morbidity and mortality among psychiatric patients is largely unknown.

In addition, comorbid illnesses are often underdiagnosed in psychiatric patients [13]. Currently, effective treatments for many chronic liver diseases exist and may reduce the risk of cirrhotic complications and HCC [14, 15]. A better understanding of the epidemiology of chronic liver diseases among patients with psychiatric illness would allow policymakers to determine the value of screening and formulate an action plan. In this project, we studied a large territory-wide Hong Kong cohort and determined the incidence of HCC and cirrhotic complications in patients with psychiatric illness.

## Methods

### Study design and data source

This is a retrospective cohort study based on data retrieved from the Clinical Data Analysis and Reporting System (CDARS) [16]. CDARS is an electronic healthcare database managed by the Hospital Authority, Hong Kong. CDARS includes in-patient and out-patient data from all public healthcare services in Hong Kong, which covers approximately 80% of the 7.4-million local population [17]. All clinical information captured in CDARS including patients’ demographic, diagnoses, procedures, drug prescription and dispensing history, and laboratory results were anonymised to ensure confidentiality. Death date and causes of death in CDARS are extracted from the death registry of the Immigration Department of Hong Kong [18]. Multiple territory-wide studies were conducted based on CDARS [19–23]. Diagnosis and procedures in CDARS were recorded by the International Classification of Diseases, Ninth Revision, Clinical Modification (ICD-9-CM) coding. The ICD-9-CM coding is validated to be 99% accurate by chart review on clinical, laboratory, imaging and endoscopy results [19]. The statistics on liver cancer stratified by age and gender of the general Hong Kong population that were available till year 2016 were extracted from the Hong Kong Cancer Registry, Hospital Authority [24]. The Hong Kong population number over the years stratified by age and gender were extracted from the Hong Kong Census and Statistics Department [25].

### Subjects

We evaluated all subjects with psychiatric illness first diagnosed between January 1, 2003 and December 31, 2007 in Hong Kong. We selected this period so that the patients would have more than 10 years of follow-up. Psychiatric illnesses were defined by ICD-9-CM diagnosis codes (290.0–319). Patients aged below 18 years old or above 70 years old at the diagnosis of psychiatric illness; had mental retardation; had dementia as the first diagnosed psychiatric illness; HCC or hepatic events before the diagnosis of psychiatric illness; and history of cancer within 5 years before the diagnosis of psychiatric illness were excluded. Patients were grouped as having mood disorders, psychotic disorders, drug-induced mental disorders, alcohol-induced mental disorders, and other psychiatric illnesses according to their diagnoses (Supplementary Table 1); patients might have more than one psychiatric diagnosis and were represented in the corresponding groups in the analysis. Patients were followed until diagnosis of liver-related event, censored at death or the last follow-up date (December 31, 2017), whichever came first. For the comparison to the Hong Kong general population on HCC incidence, patients with psychiatric illness were followed until diagnosis of HCC, censored at death or the last follow-up date (December 31, 2016), whichever came first. The study protocol was approved by the Joint Chinese University of Hong Kong - New Territories East Cluster Clinical Research Ethics Committee.

### Data collection

Data were obtained from CDARS in March 2018. We defined the baseline date as the date of first diagnosis of psychiatric illness. We collected demographic data including gender and date of birth. We also retrieved data on liver and renal biochemistries, hematological parameters, relevant diagnoses and procedures, concomitant drugs, and other laboratory parameters at baseline and during follow-up.

### Definitions

Our primary endpoint was liver-related event, which is a composite endpoint of HCC, hepatic events, and liver-related death. We defined HCC based on diagnosis codes (155.0 – hepatocellular carcinoma and 155.2 – carcinoma of liver), or procedure codes for HCC treatment (Supplementary Table 2). We defined hepatic events based on diagnosis and procedure codes of ascites, spontaneous bacterial peritonitis, variceal bleeding, hepatorenal syndrome, hepatic encephalopathy, liver transplantation, and/or liver-related death (Supplementary Table 2). Chronic hepatitis B (CHB) and chronic hepatitis C (CHC) were defined based on diagnosis codes, virological markers and the use of antiviral treatment (Supplementary Tables 3 and 4). Other liver diseases were defined based on diagnosis codes (Supplementary Table 2).

### Statistical analysis

All statistical analyses were performed using Statistical Product and Service Solutions (SPSS) version 25.0 (SPSS, Inc., Chicago, Illinois), SAS (9.4; SAS Institute Inc., Cary, NC), and R software (3.5.1; R Foundation for Statistical Computing, Vienna, Austria). Continuous variables were expressed as mean ± standard deviation or median (interquartile range [IQR]), as appropriate, while categorical variables were presented as number (percentage). Qualitative and quantitative differences between subgroups were analysed by Chi-square or Fisher’s exact tests for categorical parameters and Student’s *t* test or Mann-Whitney test for continuous parameters, as appropriate. Cumulative incidence function of HCC with adjustment of competing risk of death was estimated with 95% confidence interval (CI). Cumulative incidence function of liver-related events and liver-related death with adjustment of competing risk of death from other causes were estimated with 95% CI. Expected cumulative incidence of HCC in the general population was estimated by Ederer II method, and compared with cumulative incidence of HCC in patients with psychiatric illnesses estimated by Kaplan-Meier method. Age- and sex-standardized incidence ratio (SIR) of HCC in patients with psychiatric illnesses to the general population was estimated by Poisson model. All statistical tests were two-sided. *P* value of < 0.05 was taken as statistical significance.

## Results

### Patient characteristics

We identified 178,225 patients with psychiatric illness first diagnosed between 2003 and 2007; 72,462 were excluded according to the exclusion criteria, the vast majority due to age. Finally, 105,763 patients were included and analysed (Supplementary Figure 1). At baseline, the mean age was 43.0 ± 13.0 years; 63,614 (60.1%) were female; most patients had normal liver and renal function (Table 1). 67,964 (64.0%), 32,262 (30.5%), 10,321 (9.8%), 6066 (5.7%), 15,848 (15.0%) were diagnosed as mood disorders, psychotic disorders, drug-induced mental disorders, alcohol-induced mental disorders, and other psychiatric illnesses, respectively (Table 2); other psychiatric illnesses mainly included personality disorders, sexual disorders, sleep disorders, and dementia developing during follow-up (Supplementary Table 5).

**Table 1:** Baseline characteristics of psychiatric patients with and without liver-related events

Baseline characteristics	All	No liver-related events	Liver-related events	*P* value
N	105,763	104,302	1461	
Male gender (n, %)	42,149 (39.9)	41,186 (39.5)	963 (65.9)	< 0.001
Age (years)	43.0 ± 13.0	42.8 ± 13.0	52.6 ± 10.9	< 0.001
Platelet (× 10^9^/L)	263.5 ± 75.2	264.3 ± 74.3	217.9 ± 106.3	< 0.001
Missing (%)	27.5	27.8	11.0	
Albumin (g/L)	42.5 ± 4.6	42.6 ± 4.5	38.0 ± 6.6	< 0.001
Missing (%)	22.3	22.5	6.8	
Total bilirubin (μmol/L)	11.0 ± 8.4	10.9 ± 7.7	18.0 ± 26.8	< 0.001
Missing (%)	22.4	22.6	6.8	
Alanine aminotransferase (U/L)	18.0 (13.0–27.0)	18.0 (13.0–27.0)	30.0 (18.0–52.0)	< 0.001
Missing (%)	22.3	22.6	6.8	
Creatinine (μmol/L)	78.7 ± 56.0	77.5 ± 47.0	151.5 ± 230.6	< 0.001
Missing (%)	21.1	21.3	6.5	
Fasting glucose (mmol/L)	5.8 ± 2.1	5.8 ± 2.1	6.7 ± 3.1	< 0.001
Missing (%)	65.1	65.4	44.8	
HbA_1c_ (%)	7.0 ± 2.0	7.0 ± 2.0	7.3 ± 2.2	0.008
Missing (%)	88.3	88.6	68.7	
Total cholesterol (mmol/L)	5.1 ± 1.1	5.1 ± 1.1	4.8 ± 1.3	< 0.001
Missing (%)	67.2	67.5	48.2	
HDL cholesterol (mmol/L)	1.4 ± 0.4	1.4 ± 0.4	1.3 ± 0.5	0.010
Missing (%)	70.9	71.1	54.9	
LDL cholesterol (mmol/L)	3.1 ± 1.0	3.1 ± 1.0	2.8 ± 1.1	< 0.001
Missing (%)	71.1	71.3	55.8	
Triglyceride (mmol/L)	1.6 ± 1.5	1.6 ± 1.5	1.8 ± 1.7	0.023
Missing (%)	67.5	67.8	48.9	
Liver disease (n, %)	8256 (7.8)	7364 (7.1)	892 (61.1)	< 0.001
Chronic hepatitis B	5028 (4.8)	4578 (4.4)	450 (30.8)	< 0.001
Chronic hepatitis C	1807 (1.7)	1608 (1.5)	199 (13.6)	< 0.001
Alcohol-related liver disease	807 (0.8)	498 (0.5)	309 (21.1)	< 0.001
Fatty liver	1110 (1.0)	943 (0.9)	167 (11.4)	< 0.001
Others	207 (0.2)	169 (0.2)	38 (2.6)	< 0.001
Medication during follow-up (n, %)				
Hypnotics and Anxiolytics	70,863 (67.0)	69,835 (67.0)	1028 (70.4)	0.006
Antipsychotics	42,090 (39.8)	41,462 (39.8)	628 (43.0)	0.012
Antidepressants (n, %)				
− SNRI	8451 (8.0)	8410 (8.1)	41 (2.8)	< 0.001
− SSRI	46,438 (43.9)	45,985 (44.1)	453 (31.0)	< 0.001
− TCA	29,524 (27.9)	29,206 (28.0)	318 (21.8)	< 0.001
− Others	28,019 (26.5)	27,713 (26.6)	306 (20.9)	< 0.001
Anti-HBV treatment (n, %)^a^	1404 (27.9)	1126 (24.6)	278 (61.8)	< 0.001
Anti-HCV treatment (n, %)^b^	38 (2.1)	31 (1.9)	7 (3.5)	0.182
Follow-up duration (years)	12.4 (11.0–13.7)	12.5 (11.1–13.7)	5.3 (2.4–8.9)	< 0.001

Alanine aminotransferase and follow-up duration were expressed in median (interquartile range), whereas other continuous variables were expressed in mean ± standard deviation. Qualitative and quantitative differences between subgroups were analyzed by chi-square or Fisher’s exact tests for categorical parameters and Student’s *t* test or Mann-Whitney test for continuous parameters, as appropriate

^a^ Percentage calculated among patients with chronic hepatitis B

^b^ Percentage calculated among patients with chronic hepatitis C

*HBV* hepatitis B virus, *HCV* hepatitis C virus, *SNRI* Serotonin-norepinephrine reuptake inhibitors, *SSRI* Selective serotonin reuptake inhibitors, *TCA* Tricyclic antidepressants

**Table 2:** Clinical events in patients with different psychiatric illness

Events (n, %)	All patients	Mood disorders	Psychotic disorders	Drug-induced mental disorders	Alcohol-induced mental disorders	Other psychiatric illnesses
N	105,763	67,964	32,262	10,321	6066	15,848
Liver-related events	1461 (1.4)	701 (1.0)	614 (1.9)	180 (1.7)	348 (5.7)	228 (1.4)
- HCC	472 (0.4)	225 (0.3)	162 (0.5)	67 (0.6)	80 (1.3)	66 (0.4)
- Ascites	499 (0.5)	230 (0.3)	202 (0.6)	67 (0.6)	138 (2.3)	71 (0.4)
- Hepatic encephalopathy	352 (0.3)	152 (0.2)	189 (0.6)	52 (0.5)	137 (2.3)	64 (0.4)
- Spontaneous bacterial peritonitis	330 (0.3)	174 (0.3)	147 (0.5)	37 (0.4)	54 (0.9)	63 (0.4)
- Variceal bleeding	172 (0.2)	68 (0.1)	80 (0.2)	21 (0.2)	69 (1.1)	28 (0.2)
- Hepatorenal syndrome	58 (0.1)	21 (0.03)	26 (0.1)	5 (0.05)	21 (0.3)	12 (0.1)
- Liver transplantation	13 (0.01)	9 (0.01)	3 (0.01)	3 (0.03)	1 (0.02)	3 (0.02)
- Liver-related death	599 (0.6)	249 (0.4)	263 (0.8)	79 (0.8)	149 (2.5)	91 (0.6)
Other malignancies	5171 (4.9)	3208 (4.7)	1679 (5.2)	302 (2.9)	501 (8.3)	757 (4.8)
Overall mortality	10,614 (10.0)	5265 (7.7)	4794 (14.9)	1519 (14.7)	1213 (20.0)	1769 (11.2)

Patients might have more than one type of psychiatric illness and more than one clinical event

*HCC* hepatocellular carcinoma

### Liver disease in patients with psychiatric illness

Among 105,763 patients, 8256 (7.8%) patients had known liver diseases; 5028 (4.8%), 1807 (1.7%), 807 (0.8%), 1110 (1.0%) had CHB, CHC, alcohol-related liver disease, and fatty liver, respectively (Supplementary Table 6). Patients with drug-induced mental disorders and alcohol-induced mental disorders were more likely to have known liver diseases (18.1%); the most common liver diseases in these two groups were CHC and alcohol-related liver disease, respectively. In contrast, CHB was the most common chronic liver disease in the other groups, in line with the local epidemiology.

### Clinical outcomes of patients with psychiatric illness

**Fig. 1:**
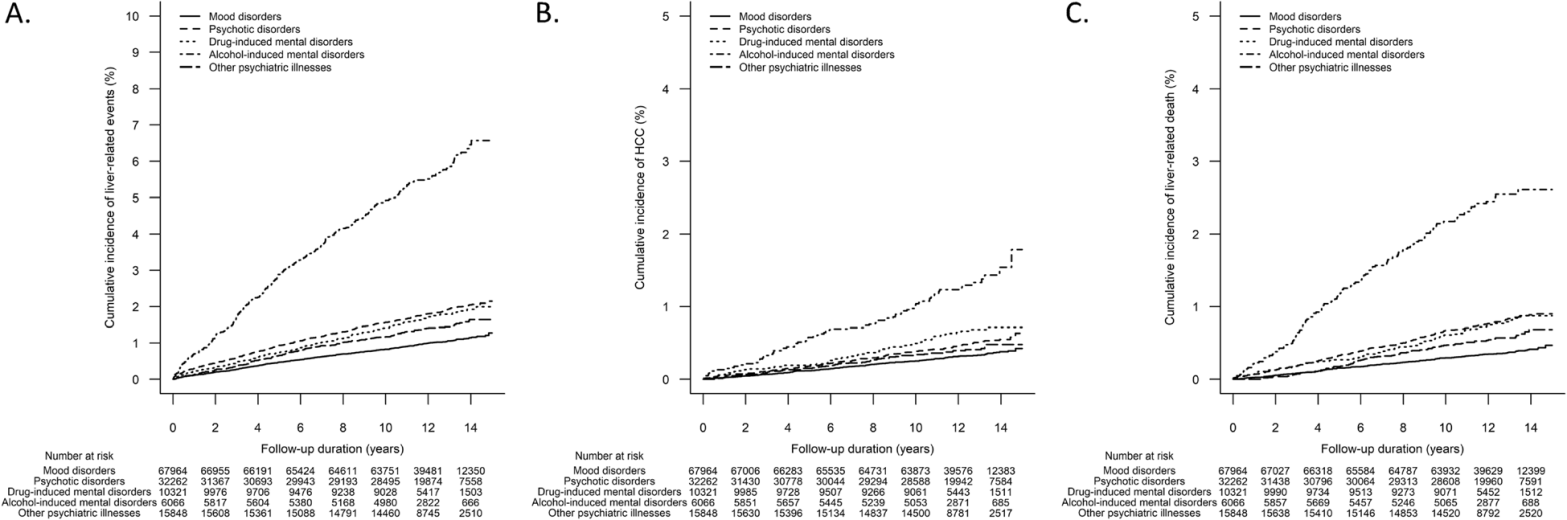
Cumulative incidence of adverse outcomes in patients with different psychiatric illnesses. **a** liver-related events, **b** hepatocellular carcinoma, **c** liver-related mortality

At a median (IQR) follow-up of 12.4 (11.0–13.7) years, 1461 (1.4%) patients developed liver-related events (Table 2). The cumulative incidence (95% CI) of liver-related events at 5, 10 and 15 years was 0.7% (0.6–0.7%), 1.1% (1.1–1.2%) and 1.6% (1.5–1.8%), respectively. Patients who had alcohol-induced mental disorders had the highest incidence of liver-related events, followed by patients with psychotic disorders and drug-induced mental disorders (Table 2). The cumulative incidence (95% CI) of liver-related events in patients with alcohol-induced mental disorders at 5, 10 and 15 years was 2.9% (2.5–3.3%), 4.9% (4.4–5.5%) and 6.6% (5.8–7.4%), respectively. The cumulative incidence (95% CI) of liver-related events in patients with psychotic disorders at 5, 10 and 15 years was 0.9% (0.8–1.0%), 1.6% (1.4–1.7%) and 2.1% (1.9–2.4%), respectively. The cumulative incidence (95% CI) of liver-related events in patients with drug-induced mental disorders at 5, 10 and 15 years was 0.7% (0.6–0.9%), 1.4% (1.2–1.7%) and 2.0% (1.7–2.3%), respectively (Fig. 1a). Among 1617 patients who developed dementia after baseline, 84 (5.2%) patients developed liver-related events (Supplementary Table 5); their mean age was 68.0 ± 8.9 years at diagnosis of dementia. Among 1461 patients who developed liver-related events, 892 (61.1%) were diagnosed with any chronic liver diseases before the event. Most patients were diagnosed with CHB, followed by alcohol-related liver disease, CHC, and fatty liver. The remaining 569 (38.9%) patients did not have any diagnosis of chronic liver diseases before liver-related events (Supplementary Table 7).

Patients who developed liver-related events were older and more likely to be male (Table 1). They also had lower baseline platelet count, albumin, total cholesterol, high-density lipoprotein and low-density lipoprotein (LDL)-cholesterol; and higher total bilirubin, alanine aminotransferase, creatinine, fasting glucose, hemoglobin A_1c_ and triglycerides. As expected, they were more likely to have known liver diseases. Patients with liver-related events were more likely to be on hypnotics and anxiolytics as well as antipsychotics, but were less likely to be on antidepressants.

Four hundred and seventy-two (0.4%) patients developed HCC (Table 2). The cumulative incidence (95% CI) of HCC at 5, 10 and 15 years was 0.2% (0.1–0.2%), 0.3% (0.3–0.4%) and 0.6% (0.5–0.6%), respectively. Patients who had alcohol-induced mental disorders had the highest HCC incidence, followed by patients with drug-induced mental disorders and psychotic disorders (Table 2). The cumulative incidence (95% CI) of HCC in patients with alcohol-induced mental disorders at 5, 10 and 15 years was 0.6% (0.4–0.8%), 1.0% (0.8–1.3%) and 1.8% (1.2–2.5%), respectively. The cumulative incidence (95% CI) of HCC in patients with drug-induced mental disorders at 5, 10 and 15 years was 0.2% (0.1–0.3%), 0.5% (0.4–0.6%) and 0.7% (0.6–0.9%), respectively. The cumulative incidence (95% CI) of HCC in patients with psychotic disorders at 5, 10 and 15 years was 0.2% (0.1–0.2%), 0.4% (0.3–0.5%) and 0.6% (0.5–0.8%), respectively (Fig. 1b).

Five hundred and ninety-nine (0.6%) patients died from liver-related causes (Table 2). The cumulative incidence (95% CI) of liver-related death at 5, 10 and 15 years was 0.2% (0.2–0.3%), 0.5% (0.4–0.5%) and 0.7% (0.6–0.7%), respectively. Patients who had alcohol-induced mental disorders had the highest occurrence of liver-related death, followed by patients with psychotic disorders and drug-induced mental disorders (Table 2). The cumulative incidence (95% CI) of liver-related death in patients with alcohol-induced mental disorders at 5, 10 and 15 years was 1.2% (0.9–1.5%), 2.2% (1.8–2.6%) and 2.6% (2.2–3.1%), respectively. The cumulative incidence (95% CI) of liver-related death in patients with psychotic disorders at 5, 10 and 15 years was 0.3% (0.3–0.4%), 0.7% (0.6–0.8%) and 0.9% (0.8–1.0%), respectively. The cumulative incidence (95% CI) of liver-related death in patients with drug-induced mental disorders at 5, 10 and 15 years was 0.3% (0.2–0.4%), 0.6% (0.5–0.8%) and 0.9% (0.7–1.1%), respectively (Fig. 1c).

### HCC incidence in patients with psychiatric illness compared to general population

**Fig. 2:**
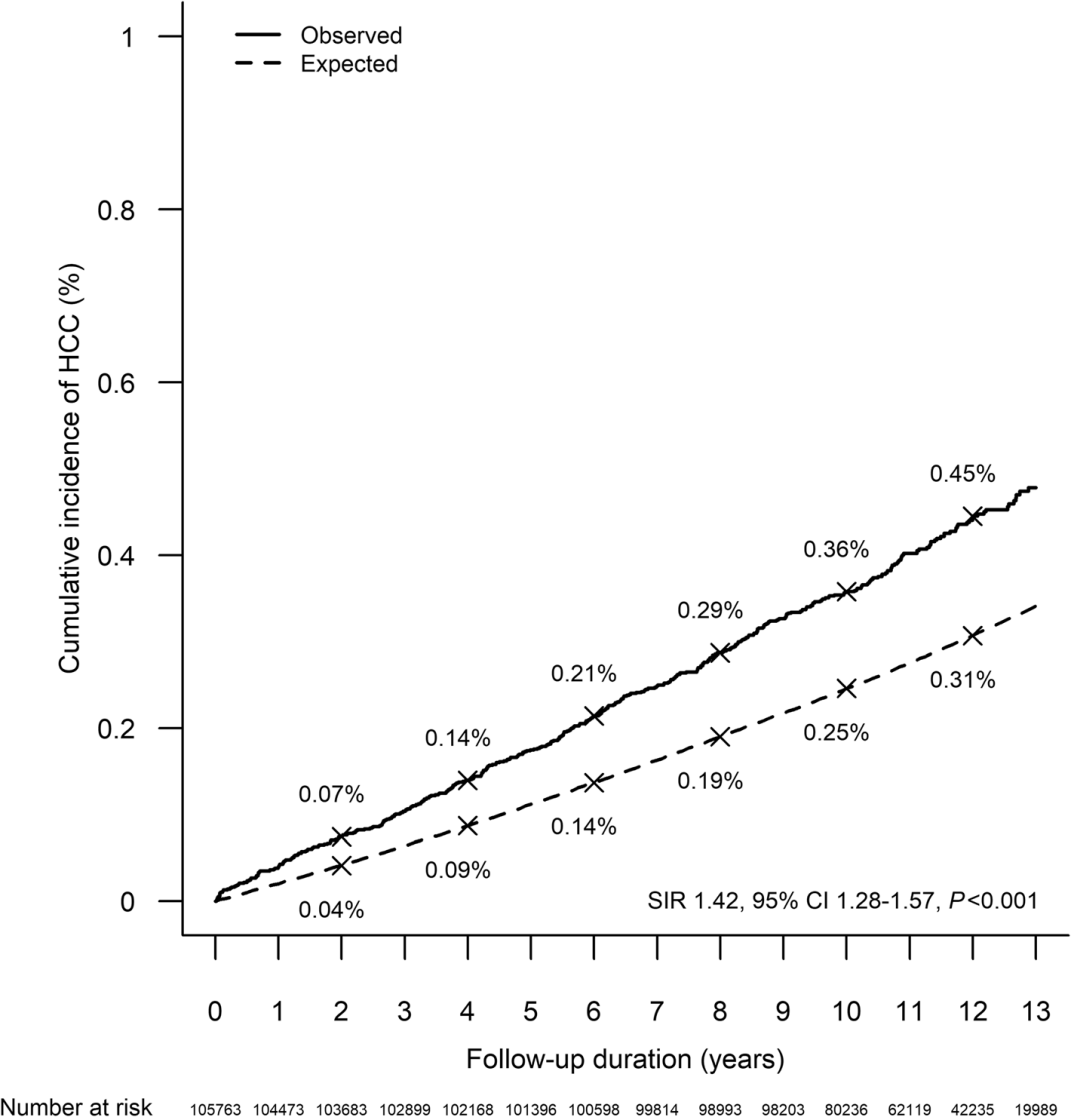
Increased incidence of hepatocellular carcinoma in patients with psychiatric illness. Expected population cumulative incidence of hepatocellular carcinoma is based on population data and observed cumulative incidence of hepatocellular carcinoma represents all patients with psychiatric illness with age- and sex-standardization

Compared to the general population, patients with psychiatric illness had a higher age- and sex-standardized incidence of HCC (SIR 1.42, 95% CI 1.28–1.57, *P* < 0.001) (Table 3 and Fig. 2). The difference in age- and sex-standardized incidence of HCC was the most significant in patients with drug-induced mental disorders (SIR 3.18, 95% CI 2.41–4.11, *P* < 0.001) and alcohol-induced mental disorders (SIR 2.98, 95% CI 2.30–3.81, *P* < 0.001) when compared to the general population (Supplementary Figure 2A and B). The difference was also significant in patients with psychotic disorders (SIR 1.39, 95% CI 1.16–1.65, *P* < 0.001) and mood disorders (SIR 1.16, 95% CI 1.00–1.34, *P* = 0.047) as compared to the general population (Table 3 and Supplementary Figure 2C and D).

**Table 3:** Age- and sex-standardized incidence ratio of hepatocellular carcinoma of patients with psychiatric illness

Psychiatric illnesses	Standardized incidence ratio^a^	95% CI	*P* value
Any psychiatric illnesses	1.42	1.28–1.57	< 0.001
Mood disorders	1.16	1.00–1.34	0.047
Psychotic disorders	1.39	1.16–1.65	< 0.001
Drug-induced mental disorders	3.18	2.41–4.11	< 0.001
Alcohol-induced mental disorders	2.98	2.30–3.81	< 0.001
Other psychiatric illnesses	1.13	0.85–1.48	0.355

Patients might have more than one type of psychiatric illness

^a^Age- and sex-standardized incidence ratio compared patients with psychiatric illness with the general Hong Kong population

*CI* confidence interval

Among 472 patients who developed HCC during follow-up, the median age at HCC diagnosis was 61 (range 26–83) years. 140 (29.7%) received curative treatment including liver resection, liver transplantation, and local ablative therapy. Sixty-four (13.6%) patients received transarterial chemoembolization, 19 (4.0%) received target therapy or immunotherapy, and 6 (1.3%) patients received chemotherapy. The remaining 243 (51.5%) patients received supportive care. Among the 472 HCC patients, 367 (77.8%) had known liver diseases. Patients with known liver disease were more likely to receive local ablative therapy than those without known liver disease (11.2% vs. 1.9%, *P* = 0.004), but there was no difference in the other treatment modalities. Overall, 30.8% of patients with known liver disease and 25.7% of patients without known liver disease received HCC treatments of curative intent (*P* = 0.315) (Table 4). The median overall survival after HCC diagnosis was 8.0 (95% CI 5.0–10.9) months.

**Table 4:** Hepatocellular carcinoma treatment in patients with psychiatric illness

Treatment	All patients	Patients with known liver disease	Patients without known liver disease	*P* value
N	472	367	105	–
Curative treatment	140 (29.7)	113 (30.8)	27 (25.7)	0.315
- Liver resection	93 (19.7)	68 (18.5)	25 (23.8)	0.230
- Liver transplantation	4 (0.8)	4 (1.1)	0 (0)	0.580
- Local ablative therapy	43 (9.1)	41 (11.2)	2 (1.9)	0.004
Transarterial chemoembolization	64 (13.6)	49 (13.4)	15 (14.3)	0.805
Target therapy or immunotherapy	19 (4.0)	16 (4.4)	3 (2.9)	0.778
Chemotherapy	6 (1.3)	4 (1.1)	2 (1.9)	0.619
Supportive care	243 (51.5)	185 (50.4)	58 (55.2)	0.383

Difference between subgroups were analyzed by chi-square or Fisher’s exact tests for categorical parameters

### Cause of death in patients with psychiatric illness

**Fig. 3:**
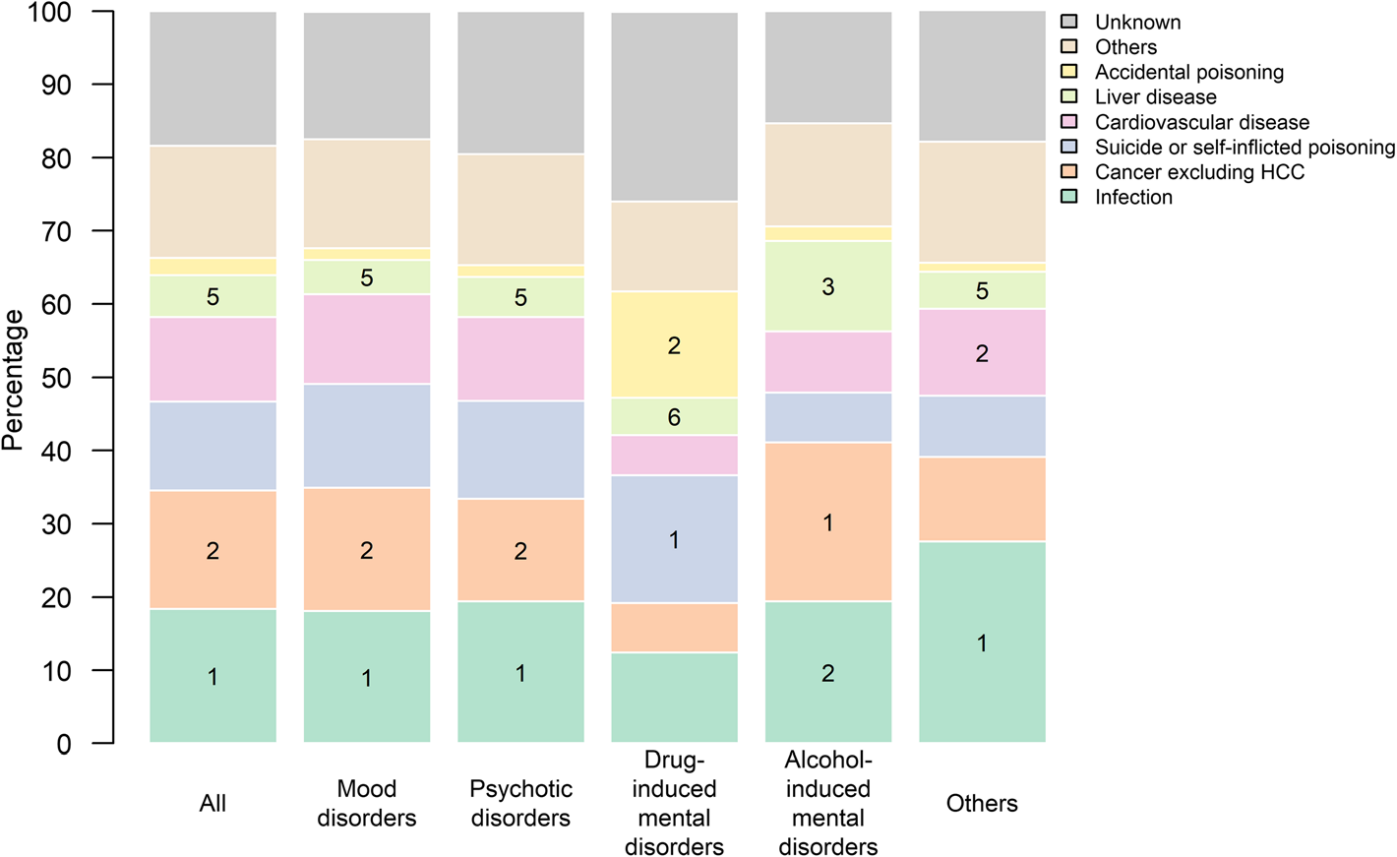
Causes of death in patients with different psychiatric illnesses. Numbers over the bars represent the ranking of the top 2 and liver-related causes of death

Among 105,763 patients, 10,614 (10.0%) patients died during follow-up (Table 2). The leading cause (percentage) of death was infection (18.4%), followed by cancers other than HCC (16.1%), suicide or self-inflicted poisoning (12.2%), and cardiovascular disease (11.5%) (Supplementary Table 8). Liver disease was the fifth leading cause of death in patients with psychiatric illness; 595 (5.6%) patients died due to liver disease (Fig. 3). Infection was the leading cause of death in patients with mood disorders, psychotic disorders, and other psychiatric illnesses, while suicide and self-inflicted poisoning was the leading cause of death in patients with drug-induced mental disorders; cancer excluding HCC was the leading cause of death in patients with alcohol-induced mental disorders. Liver disease was the fifth leading cause of death in patients with mood disorders, psychotic disorders, and other psychiatric illness, while liver disease was the third leading cause of death in patients with alcohol-induced mental disorder, and ranked the sixth in patients with drug-induced mental disorders.

## Discussion

In this large territory-wide study in Hong Kong, we demonstrate that HCC and cirrhotic complications are common in patients with psychiatric illness. Importantly, nearly 40% of patients were never diagnosed to have chronic liver diseases before the development of hepatic complications. While drug- and alcohol-induced mental disorders were associated with a 3-fold increase in HCC risk relative to the general population, the risk was also increased among patients with the common psychiatric conditions of mood disorders and psychotic disorders. Because of the low awareness of liver diseases in this population, the majority of patients with HCC could only receive supportive care.

Some psychiatric illnesses share obvious risk factors with liver diseases. In particular, 13.1% of patients with drug-induced mental disorders had documented history of CHC (Supplementary Table 6). In Hong Kong, 40–50% of injection drug users are chronically infected with hepatitis C virus [3, 26]. Although the diagnosis rate is 50% in the overall community [27], the diagnosis rate among injection drug users is much lower, and only a quarter of patients diagnosed to have CHC would eventually receive antiviral therapy. It is also noteworthy that the proportion of patients with other psychiatric illnesses who had CHC (0.9–3.3%) was still much higher than the population prevalence of positive anti-hepatitis C virus antibody (0.5%) [28]. This suggests high risk behaviours in some of the other psychiatric patients as well. Similarly, more patients with alcohol-related mental disorders had documented history of alcohol-related liver disease. The proportion of alcohol-related liver disease was relatively low about patients with other psychiatric illnesses (0.4–1.0%). The proportion of excessive alcohol use was also low in the general population [29].

As expected, the risk of HCC and cirrhotic complications was highest in patients with alcohol-induced mental disorders (5.7%) (Table 2). Nonetheless, 1.0–1.9% of the other psychiatric patients also developed hepatic complications. Although this may not be a high percentage, it is important to note that this incidence of liver-related events is much higher than what is projected from the population prevalence or incidence of advanced liver disease [29–31]. In fact, all the common psychiatric illnesses were associated with a higher risk of HCC than the general population (Table 3). Liver disease was the third leading cause of death in patients with alcohol-induced mental disorders and fifth to sixth leading cause of death in patients with other psychiatric illnesses (Fig. 3). In contrast, liver disease is not among the top ten causes of death in the general population [32]. As an analogy, a study in Minnesota suggested that liver disease was the thirteenth leading cause of death in the general population but the third leading cause of death among NAFLD patients [33]. Specific liver assessments and managements are important in high-risk populations, and our study shows an increased risk of hepatic morbidity and mortality in the psychiatric population.

If we agree that psychiatric patients represent a high-risk group for liver diseases, the next question is how to identify patients with liver disorders. The low rate of liver disease diagnosis prior to the development of liver-related complications in our study highlights the importance of improving case finding. For similar reasons, the age at HCC diagnosis was older and the overall survival after HCC diagnosis was shorter in this cohort than other reports from Hong Kong [34]. Previously, our group demonstrated the possibility to detect advanced liver disease by transient elastography in a large number of patients with type 2 diabetes [35]. Previous studies also showed that simple fibrosis scores such as the fibrosis-4 index and aspartate aminotransferase-to-platelet ratio index had high negative predictive values in excluding future liver-related events in the general population [36]. In a United Kingdom model, initial screening by the fibrosis-4 index followed by the enhanced liver fibrosis panel at the primary care setting increased the number of patients detected to have advanced fibrosis by 5 folds [37]. The alternative approach would be to detect specific liver diseases such as through screening for chronic viral hepatitis and alcohol use disorder, followed by evaluation for the severity of liver disease. At present, the implementation of methods to detect advanced liver disease in the psychiatric population has not been tested. The setting for assessment and its uptake as well as the referral pathway need to be addressed in future prospective studies. Since patients with psychiatric illnesses may be on medications that affect liver enzyme levels, the accuracy of simple fibrosis scores in such patients should be evaluated.

Before we have concrete idea about the best way to use the diagnostic tests, the current study provides important data on factors associated with liver-related events (Table 1). Male sex and older age are well known risk factors for HCC and cirrhosis [38]. Thrombocytopenia, hypoalbuminemia, hyperbilirubinemia and high alanine aminotransferase level are markers of cirrhosis and liver injury. Moreover, patients who developed liver-related events had higher fasting glucose and hemoglobin A_1c_ level at baseline. This may suggest that some patients had NAFLD [39]. Alternatively, insulin resistance is also common among patients with cirrhosis [40]. Intriguingly, patients who developed liver-related events also had lower total and LDL-cholesterol. While the exact mechanism is unclear, lipids are involved in the life cycle of hepatitis C virus [41], and eradication of hepatitis C virus is associated with a rapid increase in serum LDL-cholesterol [42].

In our study, patients who developed liver-related events were more likely to receive anxiolytics and antipsychotics but less likely to be on antidepressants (Table 1). However, the difference was confounded by indications; patients with different psychiatric illnesses were at risk of different liver diseases. Thus, the association between individual agents and liver-related outcomes was not the primary focus of this study. Previous studies showed that some antidepressants (e.g. nefazodone and trazodone) could cause serious liver injury [43]. On the other hand, a study based on the Taiwan insurance database suggests that users of tricyclic antidepressants and selective serotonin reuptake inhibitors were less likely to develop HCC [44]. While the findings are in keeping with our observation, careful assessment of confounders is required.

The association between psychiatric illness and liver-related complications is likely to be bi-directional. Some liver diseases, notably chronic hepatitis B, likely occurred before the onset of psychiatric illness. Alcohol contributes to psychiatric illness and liver disease simultaneously. NAFLD was probably aggravated by the sedentary lifestyle and obesogenic effect of drug treatments in psychiatric patients. Besides, alcohol use is common in patients with different psychiatric illnesses even though the level may not reach the definition of alcohol use disorder [45]. In NAFLD patients, modest alcohol consumption is associated with increased liver stiffness and a lower chance of histological improvement over time [29, 46].

Our study has the strength of a large sample size, long follow-up duration and the use of territory-wide data representative of the local population. Nonetheless, it also has a few limitations. First, despite the large sample size, a significant proportion of patients with psychiatric illness do not seek medical attention and would not be captured in this study [47]. The cohort is thus more representative of psychiatric patients attending hospital clinics. Second, we included patients with psychiatric diagnoses in 2003–2007, whereas the epidemiology and treatment of chronic liver diseases have changed over the years. However, we also captured treatments and interventions during follow-up until December 2017. Third, although we have laboratory data of the included patients, the database does not include non-invasive tests of liver fibrosis. A prospective study with systematic collection of fibrosis data for this special population would be informative. Fourth, a comparison of cumulative incidence of HCC among patients with psychiatric illnesses and the general population after adjusting the difference in prevalence of hepatitis viral infection and history of alcohol use was not possible due to unavailable data in the general population. In our study, the prevalence of CHC and excessive alcohol use are potentially higher among patients with psychiatric illnesses. Finally, there are notable differences in the epidemiology of liver diseases across countries. Further studies should be conducted to generate data from other regions.

## Conclusions

HCC, cirrhotic complications and liver-related deaths are common in psychiatric patients, but liver diseases are often undiagnosed. More efforts are needed to identify liver diseases in the psychiatric population so that treatments and screening for HCC and varices can be provided to patients in need.

## Supplementary information

**Additional file 1:Table S1.** ICD-9-CM diagnosis codes involved for patients with other psychiatric illnesses. **Table S2.** ICD-9-CM diagnosis and procedure codes for hepatic complications, liver cirrhosis, liver transplantation, HCC, and HCC treatment. **Table S3.** List of viral serological markers retrieved. **Table S4.** Drug codes of antiviral treatment used in Hospital Authority internally. **Table S5.** List of liver-related events for each psychiatric illness of the patients in details. **Table S6.** List of chronic liver diseases in patients with different psychiatric illness. **Table S7.** List of chronic liver diseases in 1,461 patients with liver-related events. **Table S8.** Causes of death in patients with psychiatric illness. **Figure S1.** Study patient flow. **Figure S2.** Expected cumulative incidence of hepatocellular carcinoma based on population data and observed cumulative incidence of hepatocellular carcinoma in patients with (A) drug-induced mental disorders, (B) alcohol-induced mental disorders, (C) psychotic disorders, and (D) mood disorders with age- and sex-standardization.

## Data Availability

The data that support the findings of this study are available from the Hospital Authority, Hong Kong, but restrictions apply to the availability of these data, which were used under license for the current study, and so are not publicly available. Data are however available from the authors upon reasonable request and with permission of the Hospital Authority, Hong Kong.

## Abbreviations

CDARS: Clinical Data Analysis and Reporting System
CHB: Chronic hepatitis B
CHC: Chronic hepatitis C
CI: Confidence interval
HCC: Hepatocellular carcinoma
ICD-9-CM: International Classification of Diseases, Ninth Revision, Clinical Modification
IQR: Interquartile range
LDL: Low-density lipoprotein
NAFLD: Non-alcoholic fatty liver disease
SIR: Standardized incidence ratio
