# Supplementary material for: High incidence of hepatocellular carcinoma and cirrhotic complications in patients with psychiatric illness: a territory-wide cohort study
Source: BMC Gastroenterol. 2020 Apr 29;20:128. doi: 10.1186/s12876-020-01277-0 (PMC7189713; doi:10.1186/s12876-020-01277-0)

**High incidence of hepatocellular carcinoma and cirrhotic complications in patients with psychiatric illness: A territory-wide cohort study**

**Supplementary Materials**

**Table of contents**

Supplementary Table 1…………….………………………………………..…………….…2-6

Supplementary Table 2…………….………………………………………..…………….…7-9

Supplementary Table 3…………….………..……………………………..…………………10

Supplementary Table 4…………….………..……………………………..…………………11

Supplementary Table 5…………….………..……………………………..…………………12

Supplementary Table 6…………….………..……………………………..…………………13

Supplementary Table 7…………….………..……………………………..…………………14

Supplementary Table 8…………….………..……………………………..…………………15

Supplementary Figure 1………….………..……………………………..…………………...16

Supplementary Figure 2.…………….………..……………………………..……………17-18

Supplementary Table 1. ICD-9-CM diagnosis codes involved for patients with other psychiatric illnesses.

| ICD-9-CM diagnosis code | Description |
| --- | --- |
| 290.0* | Senile dementia, uncomplicated |
| 290.10* | Presenile dementia |
| 290.40* | Arteriosclerotic dementia NOS |
| 294.01 | Post-traumatic amnesia <25hr |
| 294.02 | Post-traumatic amnesia >24h<15d |
| 294.03 | Post-traumatic amnesia >14d |
| 294.08 | Post-traumatic amnesia NOS |
| 294.09 | Amnestic syndrome, NOS |
| 294.1* | Dementia in other diseases |
| 294.8* | Organic brain syndrome NEC |
| 294.9* | Organic brain syndrome NOS |
| 299.00 | Infantile autism-current or active state |
| 299.01 | Infantile autism-residual state |
| 299.10 | Childhood disintegrative disorder, current or active state |
| 299.80 | Pervasive developmental disorders NEC-active |
| 299.90 | Pervasive developmental disorders NOS-active |
| 300.11 | Conversion disorder |
| 300.12 | Psychogenic amnesia |
| 300.13 | Psychogenic fugue |
| 300.14 | Multiple personality |
| 300.15 | Dissociative reaction NOS |
| 300.16 | Factitious illness with symptom |
| 300.19 | Factitious illness NEC/NOS |
| 300.5 | Neurasthenia |
| 300.6 | Depersonalization syndrome |
| 300.7 | Hypochondriasis |
| 300.81 | Somatization disorder |
| 300.82 | Undifferentiated somatoform disorder |
| 301 | Personality disorders |
| 301.0 | Paranoid personality |
| 301.11 | Chronic hypomanic person |
| 301.13 | Cyclothymic disorder |
| 301.20 | Schizoid personality NOS |
| 301.21 | Introverted personality |
| 301.22 | Schizotypal personality |
| 301.3 | Explosive personality |
| 301.50 | Histrionic person NOS |
| 301.51 | Chronic factitious illness |
| 301.59 | Histrionic person NEC |
| 301.6 | Dependent personality |
| 301.7 | Antisocial personality |
| 301.81 | Narcissistic personality |
| 301.82 | Avoidant personality |
| 301.83 | Borderline personality |
| 301.84 | Passive-aggressive person |
| 301.89 | Personality disorder NEC |
| 301.9 | Personality disorder NOS |
| 302 | Sexual disorders |
| 302.0 | Homosexuality |
| 302.2 | Pedophilia |
| 302.3 | Transvestism |
| 302.4 | Exhibitionism |
| 302.50 | Trans-sexualism NOS |
| 302.51 | Trans-sexualism, asexual |
| 302.53 | Trans-sex, heterosexual |
| 302.6 | Psychosexual identity disorder |
| 302.70 | Psychosexual dysfunction NOS |
| 302.71 | Inhibited sexual desire |
| 302.72 | Inhibited sex excitement |
| 302.73 | Inhibited female orgasm |
| 302.74 | Inhibited male orgasm |
| 302.75 | Premature ejaculation |
| 302.76 | Functional dyspareunia |
| 302.79 | Psychosexual dysfunction NEC |
| 302.81 | Fetishism |
| 302.82 | Voyeurism |
| 302.83 | Sexual masochism |
| 302.84 | Sexual sadism |
| 302.85 | Gender identity disorder, adult |
| 302.9 | Psychosexual disorder NOS |
| 306 | Psychophysiologic disorder |
| 306.0 | Psychogenic musculoskeletal disorder |
| 306.1 | Psychogenic respiratory disease |
| 306.2 | Psychogenic cardiovascular disease |
| 306.3 | Psychogenic skin disease |
| 306.4 | Psychogenic gastrointestinal disorder |
| 306.50 | Psychogenic genitourinary disorder NOS |
| 306.51 | Psychogenic vaginismus |
| 306.52 | Psychogenic dysmenorrhea |
| 306.53 | Psychogenic dysuria |
| 306.7 | Psychogenic sensory disorder |
| 306.8 | Psychogenic disorder NEC |
| 306.9 | Psychogenic disorder NOS |
| 307 | Special symptom NEC |
| 307.0 | Stammering & stuttering |
| 307.1 | Anorexia nervosa |
| 307.20 | Tic disorder NOS |
| 307.22 | Chronic motor tic disorder |
| 307.23 | Gilles Tourette disorder |
| 307.3 | Stereotyped movements |
| 307.40 | Nonorganic sleep dis NOS |
| 307.42 | Persistent insomnia |
| 307.44 | Persistent hypersomnia |
| 307.45 | Disrupt sleep-wake cycle |
| 307.46 | Somnambulism/night terror |
| 307.47 | Sleep stage dysfunction NEC |
| 307.49 | Nonorganic sleep disorder NEC |
| 307.50 | Eating disorder NOS |
| 307.51 | Bulimia |
| 307.52 | Pica |
| 307.53 | Psychogenic rumination |
| 307.54 | Psychogenic vomiting |
| 307.59 | Eating disorder NEC |
| 307.6 | Enuresis, nonorganic |
| 307.7 | Encopresis |
| 307.80 | Psychogenic pain NOS |
| 307.81 | Tension headache |
| 307.89 | Psychogenic pain NEC |
| 307.9 | Special symptom NEC/NOS |
| 310 | Nonpsychotic brain syndrome |
| 310.0 | Frontal lobe syndrome |
| 310.1 | Organic personality syndrome |
| 310.2 | Postconcussion syndrome |
| 310.8 | Nonpsychotic brain syndrome NEC |
| 310.9 | Nonpsychotic brain syndrome NOS |
| 312 | Conduct disturbance |
| 312.00 | Undersocialized conduct disorder, aggressive, unspecified |
| 312.01 | Undersocialized conduct disorder, aggressive, unspecified |
| 312.02 | Undersocialized conduct disorder, aggressive, moderate |
| 312.03 | Undersocialized conduct disorder, aggressive, severe |
| 312.10 | Undersocialized conduct disorder, unaggressive, unspecified |
| 312.12 | Undersocialized conduct disorder, unaggressive, moderate |
| 312.20 | Socialized conduct disorder, unspecified |
| 312.21 | Socialized conduct disorder, mild |
| 312.22 | Socialized conduct disorder, moderate |
| 312.23 | Socialized conduct disorder, severe |
| 312.30 | Impulse control disorder NOS |
| 312.31 | Pathological gambling |
| 312.32 | Kleptomania |
| 312.33 | Pyromania |
| 312.34 | Intermittent explosive disorder |
| 312.35 | Isolated explosive disorder |
| 312.39 | Impulse control disorder NEC |
| 312.4 | Mixed disturbance of conduct and emotions |
| 312.81 | Conduct disorder, childhood onset type |
| 312.82 | Conduct disorder, adolescent onset type |
| 312.89 | Other conduct disturbance |
| 312.9 | Conduct disturbance NOS |
| 313.23 | Elective mutism |
| 313.3 | Relationship problems |
| 313.81 | Oppositional disorder |
| 313.82 | Identity disorder |
| 313.83 | Academic underachievement |
| 314.00 | Attention deficit disorder without mention of hyperactivity |
| 314.01 | Attention deficit disorder without hyperactivity |
| 314.1 | Hyperkinesis with developmental delay |
| 314.2 | Hyperkinetic conduct disorder |
| 314.8 | other hyperkinetic syndrome |
| 314.9 | hyperkinetic syndrome NOS |
| 315 | Specific delays in development |
| 315.00 | Reading disorder NOS |
| 315.01 | Alexia |
| 315.02 | Developmental dyslexia |
| 315.09 | Reading disorder NEC |
| 315.1 | Arithmetical disorder |
| 315.2 | Other learning difficulty |
| 315.31 | Expressive language disorder |
| 315.32 | Receptive-expressive language disorder |
| 315.39 | Speech/language disorder NEC |
| 315.4 | Coordination disorder |
| 315.5 | Mixed development disorder |
| 315.8 | Development delays NEC |
| 315.9 | Development delays NOS |
| 316 | Psychic factors associated with diseases classified elsewhere |

*Other mental illnesses included dementia developed during follow-up.

NEC = not elsewhere classified, NOS = not otherwise specified.

Supplementary Table 2. ICD-9-CM diagnosis and procedure codes for hepatic complications, liver cirrhosis, liver transplantation, HCC, and HCC treatment.

| Disease | ICD-9-CM Code | Description |
| --- | --- | --- |
| Viral Hepatitis |  |  |
| Chronic hepatitis B | 070.22 | Chronic viral hepatitis B with hepatic coma without hepatitis delta |
| Chronic hepatitis B | 070.23 | Chronic viral hepatitis B with hepatic coma with hepatitis delta |
| Chronic hepatitis B | 070.32 | Chronic viral hepatitis B without mention of hepatic coma without mention of hepatitis delta |
| Chronic hepatitis B | 070.33 | Chronic viral hepatitis B without mention of hepatic coma with hepatitis delta |
| Chronic hepatitis B | V02.61 | Hepatitis B carrier |
| Acute hepatitis B | 070.20 | Viral hepatitis B with hepatic coma, acute or unspecified, without mention of hepatitis delta |
| Acute hepatitis B | 070.21 | Viral hepatitis B with hepatic coma, acute or unspecified, with hepatitis delta |
| Acute hepatitis B | 070.30 | Viral hepatitis B without mention of hepatic coma, acute or unspecified, without mention of hepatitis delta |
| Acute hepatitis B | 070.31 | Viral hepatitis B without mention of hepatic coma, acute or unspecified, with hepatitis delta |
| Hepatitis C | 070.41 | Acute hepatitis C with hepatic coma |
| Hepatitis C | 070.44 | Chronic hepatitis C with hepatic coma |
| Hepatitis C | 070.51 | Acute hepatitis C without mention of hepatic coma |
| Hepatitis C | 070.54 | Chronic hepatitis C without mention of hepatic coma |
| Hepatitis C | V02.62 | Hepatitis C carrier |
| Hepatitis D | 070.23 | Chronic viral hepatitis B with hepatic coma with hepatitis delta |
| Hepatitis D | 070.33 | Chronic viral hepatitis B without mention of hepatic coma with hepatitis delta |
| Hepatitis D | 070.42 | Hepatitis delta without mention of active hepatitis B disease with hepatic coma |
| Hepatitis D | 070.52 | Hepatitis delta without mention of active hepatitis B disease or hepatic coma |
| Other liver disease | | |
| Alcoholic liver disease | 571.0 | Alcoholic fatty liver |
| Alcoholic liver disease | 571.1 | Acute alcoholic hepatitis |
| Alcoholic liver disease | 571.2 | Alcoholic cirrhosis of live |
| Alcoholic liver disease | 571.3 | Alcoholic liver damage, unspecified |
| Fatty liver | 571.5:4, 571.51 | Non-alcoholic cirrhosis - A |
| Fatty liver | 571.4:5, 571.52 | Non-alcoholic cirrhosis - B |
| Fatty liver | 571.4:6, 571.53 | Non-alcoholic cirrhosis - C |
| Fatty liver | 571.8:1 | Fatty liver |
| Fatty liver | 571.49:2, 571.8:4 | Steatohepatitis |
| Primary biliary cholangitis | 571.6:1 | Primary biliary cholangitis |
| Wilson’s disease | 275.1:0 | Disorders of copper metabolism |
| Wilson’s disease | 275.1:2 | Wilson’s disease |
| Autoimmune hepatitis | 571.42 | Autoimmune hepatitis |
| Hepatic complications |  |  |
| Ascites | 789.5 | Ascites |
| SBP | 567.2:9 | Spontaneous bacterial peritonitis |
| SBP | 567.8:0 | Peritonitis |
| EVB^a^ | 456.0 | Oesophageal varices with bleeding |
| EVB | 456.20 | Oesophageal varices classified elsewhere with bleeding |
| GVB^a^ | 456.8:1 | Fundal varices, bleeding |
| GVB | 456.8:2 | Bleeding gastric varices |
| HE | 348.3 | Encephalopathy, unspecified |
| HE | 349.82 | Toxic encephalopathy |
| HE | 572.2 | Hepatic coma |
| HRS | 572.4 | Hepatorenal syndrome |
| Portal hypertension | 572.3 | Portal hypertension |
| Varices | 456.1 | Oesophageal varices without bleeding |
| Varices | 456.21 | Oesophageal varices in diseases classified elsewhere without bleeding |
| Varices | 456.8:4 | Fundal varices |
| Varices | 456.8:5 | Gastric varices |
| Liver cirrhosis |  |  |
| Liver cirrhosis | 571.2 | Alcoholic cirrhosis of liver |
| Liver cirrhosis | 571.5 | Cirrhosis of liver without mention of alcohol |
| Liver transplantation |  |  |
| Liver transplantation | V42.7 | Liver replaced by transplant |
| Liver transplantation | 50.51 | Auxiliary liver transplant |
| Liver transplantation | 50.59 | Other transplant of liver |
| Hepatocellular carcinoma (HCC)^b^ | | |
| HCC | 155.0 | Malignant neoplasm of liver, primary |
| HCC | 155.2 | Malignant neoplasm of liver, not specified |
| HCC treatment ^b^ | | |
| Liver resection | 50.22 | Partial hepatectomy |
| Liver resection | 50.3 | Hepatic lobectomy |
| Liver resection | 50.4 | Total hepatectomy |
| Liver resection | 50.99 | Liver operation NEC |
| Radiofrequency ablation | 50.29 | Other destruction of lesion of liver |
| Percutaneous ethanol injection | 50.94 | Other injection of therapeutic substance into liver |
| TOCE | 38:80:4 | Transarterial oily chemoembolization |
| TOCE | 99.25:2 | Transarterial oily chemoembolization |
| TAE | 38.80:3 | Transarterial embolization |
| TACE | 38.80:8 | Transcatheter arterial chemoembolization |
| Chemotherapy | 99.25 | Chemotherapy |

^a^ Oesophageal or gastric variceal bleeding was also defined by the ICD-9-CM procedure codes of 42.33:3, 42.33:6, 42.33:13, and 43.41:1

^b^ The use of sorafenib was identified from drug dispensing history

Abbreviations: EVB = oesophageal variceal bleeding; GVB = gastric variceal bleeding, HCC = hepatocellular carcinoma, HE = hepatic encephalopathy, HRS = hepatorenal syndrome, ICD-9-CM = International Classification of Diseases, Ninth Revision, Clinical Modification, SBP = spontaneous bacterial peritonitis.

| **HBV** | **HCV** | **HDV** |
| --- | --- | --- |
| HBsAg | Anti-HCV | Anti-HDV |
| HBeAg | HCV RNA (viral load), RT-PCR |  |
| Anti-HBe | HCV RNA, RT-PCR |  |
| HBV DNA |  |  |
| HBV DNA (viral load), RT-PCR |  |  |
| Anti-HBs |  |  |
| Anti-HBs, Quantitative |  |  |

Supplementary Table 3. List of viral serological markers retrieved.

Anti-HBe = antibody to hepatitis B e antigen; Anti-HBs = antibody to hepatitis B surface antigen; HBeAg = hepatitis B e antigen; HBsAg = hepatitis B surface antigen; HBV = hepatitis B virus; HCV = hepatitis C virus; HDV = hepatitis D virus; RT-PCR = Reverse transcription polymerase chain reaction.

Supplementary Table 4. Drug codes of antiviral treatment used in Hospital Authority internally.

| Drug code | Name | Dosage |
| --- | --- | --- |
| ADEF01 | Adefovir Dipivoxil | 10 MG |
| ENTE01 | Entecavir | 0.5 MG |
| ENTE02 | Entecavir | 1.0 MG |
| INTE04/05/18/19 | Interferon alpha-2a | 3-9MIU/0.5-1ML |
| INTE06-09/16-17 | Interferon alpha-2b | 3/5/10/15/25 MIU/1ML |
| LAMI07 | Lamivudine | 150 MG |
| LAMI08 | Lamivudine Solution | 10 MG/ML |
| LAMI09 | Lamivudine | 100 MG |
| LAMI10 | Lamivudine + Zidovudine | 150 MG/300 MG |
| PEGI01-03/05/09-12/18-21 | Peginterferon alpha-2b | 50-120 MCG/0.5-1ML |
| PEGI04/06-08/13/15-17 | Peginterferon alpha-2a | 135-180 MCG/0.5-1ML |
| PEGI14 | Peginterferon lambda-1a | 180 MCG/0.45ML |
| TELB01 | Telbivudine | 600 MG |
| TENO03/04/07 | Tenofovir Disoproxil Fumarate | 300 MG |
| TENO06/08 | Tenofovir Alafenamide | 25 MG |
| ASUN01 | Asunaprevir | 100MG |
| BOCE01/02 | Boceprevir | 200MG |
| DACL03 | Daclatasvir | 60MG |
| DASA08 | Dasabuvir | 250MG |
| EPCL01 | Sofosbuvir + Velpatasvir | 400MG/100MG |
| HARV01 | Ledipasvir + Sofosbuvir. | 90MG/400MG |
| MAVI01 | Glecaprevir + Pibrentasvir | 100MG/40MG |
| SOFO01 | Sofosbuvir | 400MG |
| VIEK01 | Ombitasvir + Paritaprevir + Ritonavir & Dasabuvir | 12.5MG/75MG/50MG & 250MG |
| ZEPA01 | Elbasvir + Grazoprevir | 50MG/100MG |

Supplementary Table 5. List of liver-related events for each psychiatric illness of the patients in details.

| **Psychiatric illnesses*** | **Number of patients (% in cohort)** | **Number of liver-related events**  **(% in the patients with illness)** |
| --- | --- | --- |
| Mood disorders | 67,964 (64.3) | 701 (1.0) |
| Psychotic disorders | 32,262 (30.5) | 614 (1.9) |
| Drug-induced mental disorders | 10,321 (9.8) | 180 (1.7) |
| Alcohol-induced mental disorders | 6,066 (5.7) | 348 (5.7) |
| Personality or sexual disorders | 4,175 (3.9) | 52 (1.2) |
| Sleep disorders | 2,300 (2.2) | 31 (1.3) |
| Dementia after baseline | 1,617 (1.5) | 84 (5.2) |
| Conversion/Factitious/Dissociative/  Somatoform disorders | 1,170 (1.1) | 12 (1.0) |
| Physiological malfunction arising from mental factors | 850 (0.8) | 4 (0.5) |
| Eating disorders | 466 (0.4) | 2 (0.4) |
| Pervasive developmental disorder | 352 (0.3) | 2 (0.6) |
| Amnesia | 241 (0.2) | 10 (4.1) |
| Other psychiatric illnesses | 5,672 (5.4) | 54 (1.0) |

*The psychiatric illnesses of the patient are not mutually exclusive, *i.e.* patients with liver-related events may have more than one psychiatric illness.

Supplementary Table 6. List of chronic liver diseases in patients with different psychiatric illness.

| Events (n, %) | All patients | Mood disorders | Psychotic disorders | Drug-induced mental disorders | Alcohol-induced mental disorders | Other psychiatric illnesses |
| --- | --- | --- | --- | --- | --- | --- |
| N | 105,763 | 67,964 | 32,262 | 10,321 | 6,066 | 15,848 |
| Liver disease (n, %) | 8,256 (7.8) | 4,793 (7.1) | 2,436 (7.6) | 1,865 (18.1) | 1,098 (18.1) | 1,252 (7.9) |
| - Chronic hepatitis B | 5,028 (4.8) | 3,327 (4.9) | 1,375 (4.3) | 575 (5.6) | 361 (6.0) | 779 (4.9) |
| - Chronic hepatitis C | 1,807 (1.7) | 613 (0.9) | 591 (1.8) | 1,356 (13.1) | 200 (3.3) | 213 (1.3) |
| - Alcohol-related liver disease | 807 (0.8) | 247 (0.4) | 334 (1.0) | 89 (0.9) | 665 (11.0) | 123 (0.8) |
| - Fatty liver | 1,110 (1.0) | 792 (1.2) | 307 (1.0) | 97 (0.9) | 0 (0) | 222 (1.4) |
| - Others | 207 (0.2) | 133 (0.2) | 72 (0.2) | 14 (0.1) | 21 (0.3) | 26 (0.2) |

Patients might have more than one type of liver disease.

Supplementary Table 7. List of chronic liver diseases in 1,461 patients with liver-related events.

| **Chronic liver diseases*** | **Number of patients (%)** |
| --- | --- |
| **Any chronic liver disease(s)** | 892 (61.1) |
| - Hepatitis B | 266 (18.2) |
| - Hepatitis C | 99 (6.8) |
| - Alcoholic liver disease | 206 (14.1) |
| - Fatty liver | 31 (2.1) |
| - Wilson’s disease | 0 (0) |
| - Autoimmune hepatitis | 0 (0) |
| - Primary biliary cholangitis | 2 (0.1) |
| - Hepatitis B/Hepatitis C | 19 (1.3) |
| - Hepatitis B/Alcoholic liver disease | 67 (4.6) |
| - Hepatitis B/Fatty liver | 86 (5.9) |
| - Hepatitis C/Alcoholic liver disease | 30 (2.1) |
| - Hepatitis C/Fatty liver | 38 (2.6) |
| - Primary biliary cholangitis/Fatty liver | 5 (0.3) |
| - Hepatitis B/Hepatitis C/Alcoholic liver disease | 5 (0.3) |
| - Hepatitis B/Hepatitis C/Fatty liver | 7 (0.5) |
| - Hepatitis C/Alcoholic liver disease/Recurrent pyogenic cholangitis | 1 (0.1) |
| - Unspecified chronic liver disease | 30 (2.1) |
| **Without any chronic liver diseases** | 569 (38.9) |

*The aetiologies of chronic liver disease are mutually exclusive.

Supplementary Table 8. Causes of death in patients with psychiatric illness.

| **Aetiology** | **Number of patients (%) N=10,614** |
| --- | --- |
| **Infection** | **1,956 (18.4)** |
| - Pneumonia | 1,705 (16.1) |
| - Sepsis | 251 (2.4) |
| **Cancers other than HCC** | **1,708 (16.1)** |
| - Digestive organs and peritoneum | 507 (4.8) |
| - Respiratory and intrathoracic organs | 490 (4.6) |
| - Genitourinary organs | 150 (1.4) |
| - Bone, connective tissue, skin, and breast | 129 (1.2) |
| - Others | 347 (3.3) |
| - Secondary cancer | 85 (0.8) |
| **Suicide or self-inflicted poisoning** | **1,294 (12.2)** |
| **Cardiovascular disease** | **1,221 (11.5)** |
| - Ischemic heart disease | 496 (4.7) |
| - Cerebrovascular accident | 462 (4.4) |
| - Congestive heart failure | 263 (2.5) |
| **Liver disease** | **595 (5.6)** |
| - Hepatic events | 292 (2.8) |
| - HCC | 219 (2.1) |
| - Liver failure | 86 (0.8) |
| **Accidental poisoning** | **260 (2.4)** |
| **Obstructive lung disease** | **160 (1.5)** |
| **Renal failure** | **139 (1.3)** |
| **Others** | **1,327 (12.2)** |
| **Unknown** | **1,954 (18.4)** |

Supplementary Figure 1. Study patient flow.


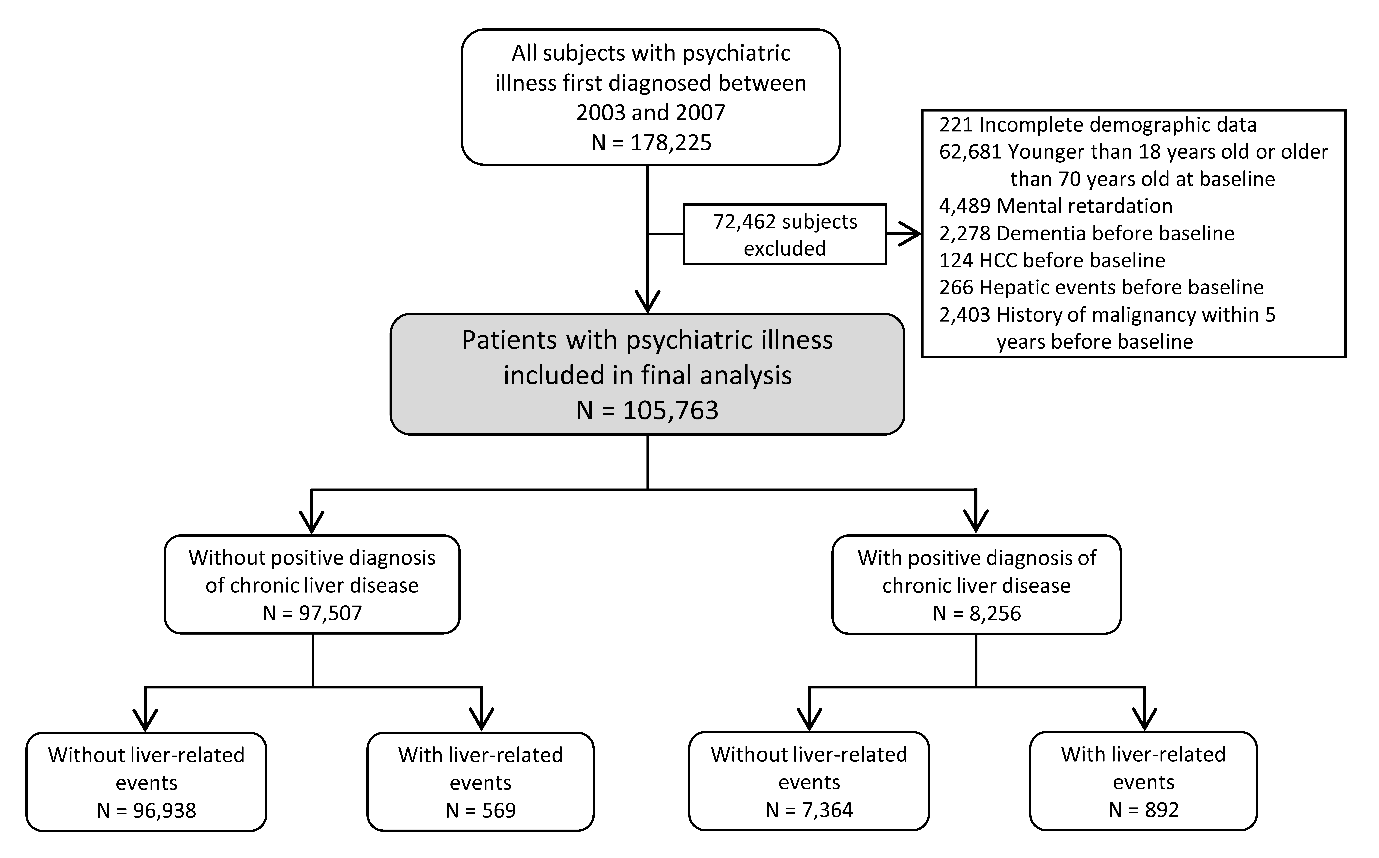


Supplementary Figure 2. Expected cumulative incidence of hepatocellular carcinoma based on population data and observed cumulative incidence of hepatocellular carcinoma in patients with (A) drug-induced mental disorders, (B) alcohol-induced mental disorders, (C) psychotic disorders, and (D) mood disorders with age- and sex-standardization.

A.


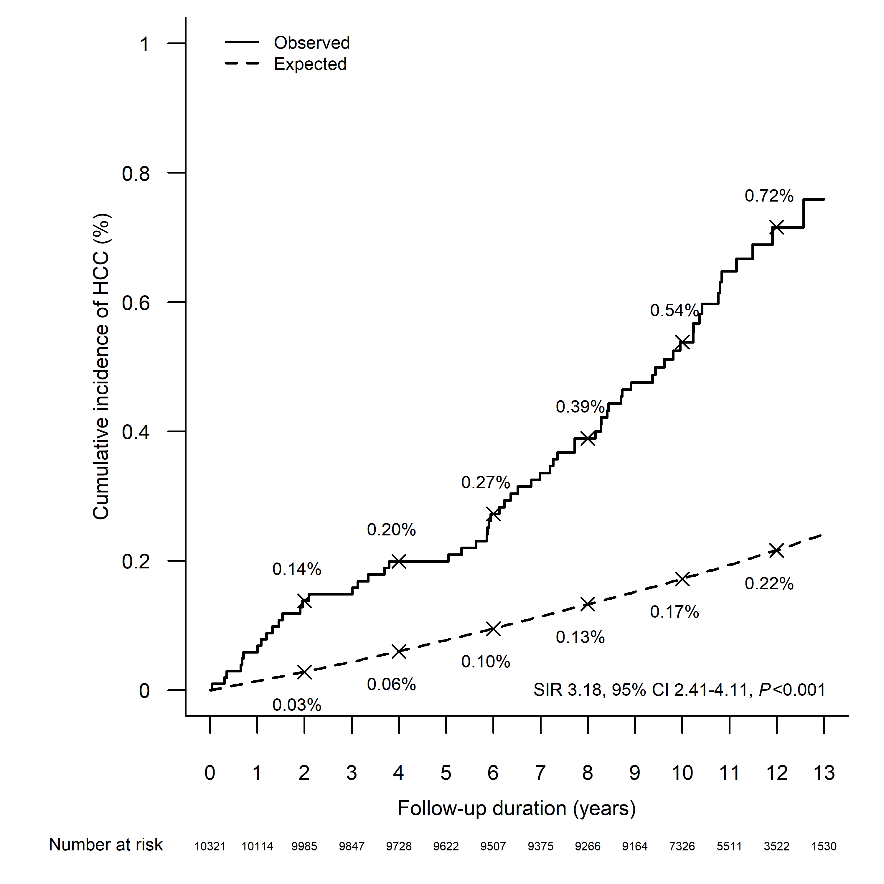


B.


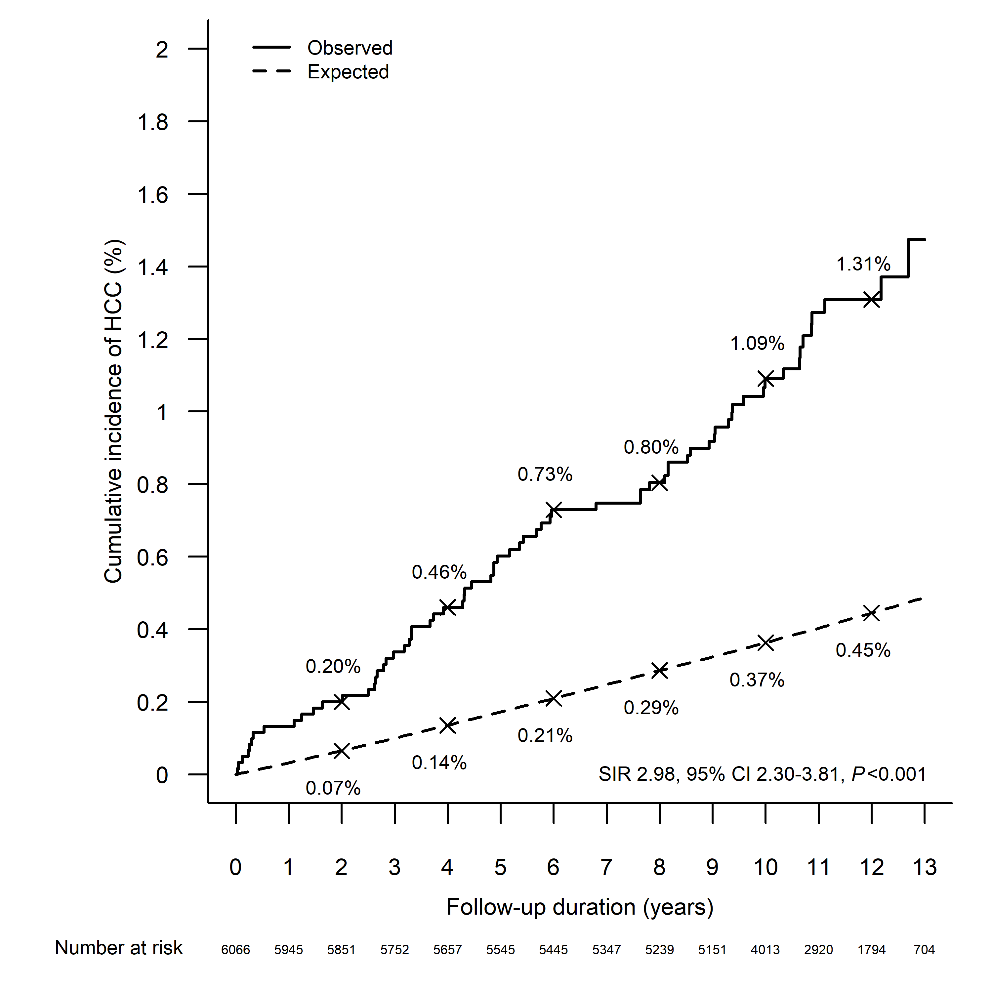


C.


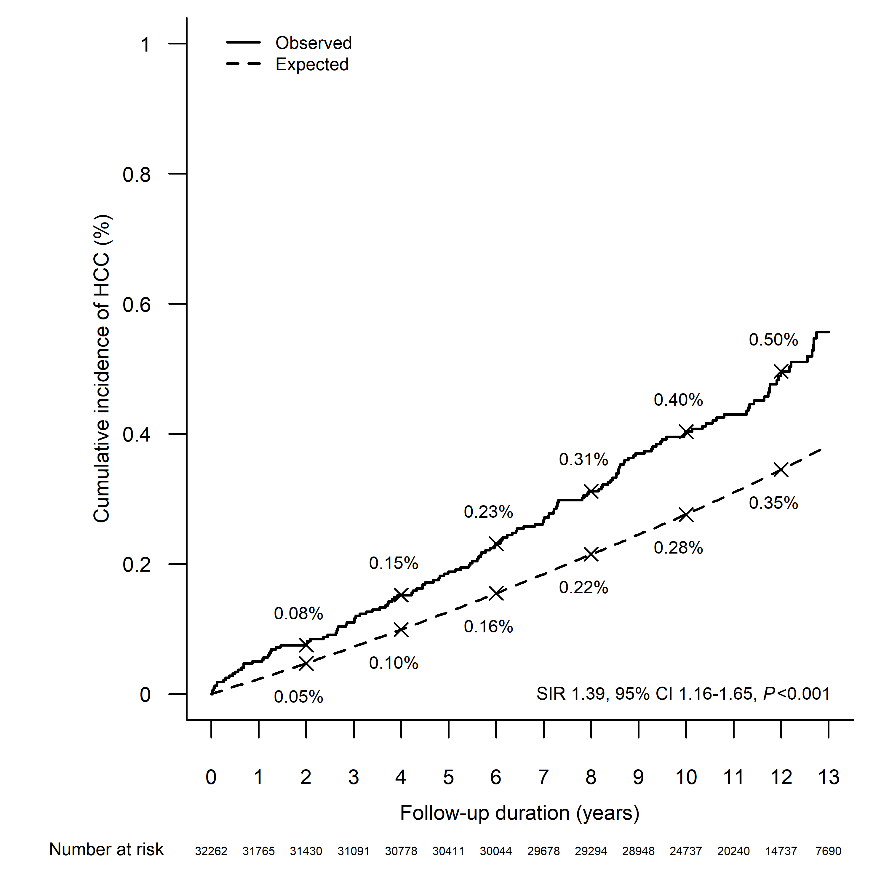


D.


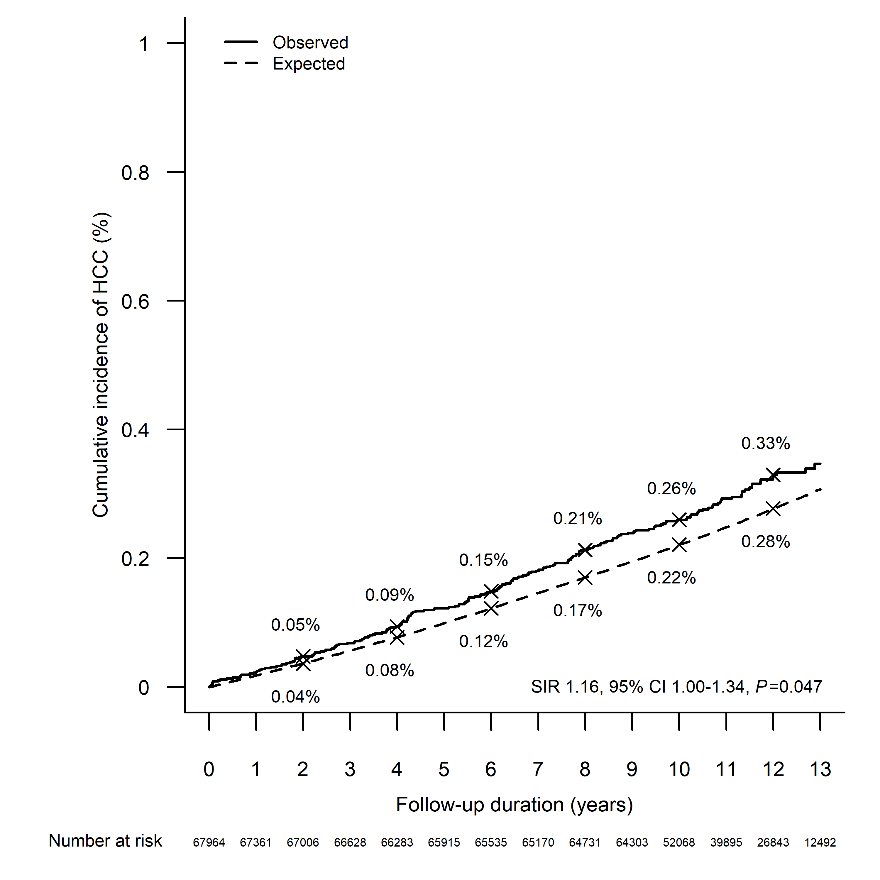

Supplement: Supplementary file 1 — Additional file 1:Table S1. ICD-9-CM diagnosis codes involved for patients with other psychiatric illnesses. Table S2. ICD-9-CM diagnosis and procedure codes for hepatic complications, liver cirrhosis, liver transplantation, HCC, and HCC treatment. Table S3. List of viral serological markers retrieved. Table S4. Drug codes of antiviral treatment used in Hospital Authority internally. Table S5. List of liver-related events for each psychiatric illness of the patients in details. Table S6. List of chronic liver diseases in patients with different psychiatric illness. Table S7. List of chronic liver diseases in 1,461 patients with liver-related events. Table S8. Causes of death in patients with psychiatric illness. Figure S1. Study patient flow. Figure S2. Expected cumulative incidence of hepatocellular carcinoma based on population data and observed cumulative incidence of hepatocellular carcinoma in patients with (A) drug-induced mental disorders, (B) alcohol-induced mental disorders, (C) psychotic disorders, and (D) mood disorders with age- and sex-standardization. [file 12876_2020_1277_MOESM1_ESM.docx]
